# Sedentary Work in Desk-Dominated Environments: A Data-Driven Intervention Using Intervention Mapping

**DOI:** 10.2196/14951

**Published:** 2020-07-20

**Authors:** Nathalie M Berninger, Gill A ten Hoor, Guy Plasqui, Gerjo Kok, Gjalt-Jorn Ygram Peters, Robert A C Ruiter

**Affiliations:** 1 Department of Work and Social Psychology Maastricht University Maastricht Netherlands; 2 Department of Nutrition and Movement Sciences Maastricht University Medical Centre Maastricht Netherlands; 3 Faculty of Psychology and Education Science Open University of The Netherlands Heerlen Netherlands

**Keywords:** intervention mapping, sedentary behavior, sedentary work, computer-based, occupational health, eHealth, mHealth, data-driven programs

## Abstract

**Background:**

Since desk-dominated work environments facilitate sedentary behavior, office workers sit for 66% of their working days and only 8% succeed in interrupting their prolonged periods of sitting within the first 55 minutes. Yet stretches of long and uninterrupted sitting increase the likelihood of several chronic metabolic and cardiovascular diseases.

**Objective:**

We therefore developed a computer-based app designed to interrupt periods of prolonged sitting among office employees.

**Methods:**

When developing the intervention, we applied the intervention mapping protocol. This approach for the systematic design of theory and evidence-based behavior change programs consists of 6 steps: creation of a logic model of the problem, creation of a logic model of change, program design, program production, design of an implementation plan, and development of an evaluation plan.

**Results:**

Working through all 6 steps has resulted in an individually adaptable intervention to reduce sedentary behavior at work. The intervention, UPcomplish, consists of tailored, half-automatized motivational components delivered by a coach. To register sedentary behavior, the VitaBit (VitaBit Software International BV) toolkit, a wearable accelerometry-based monitoring device, is used. Among others, UPcomplish includes personalized goal setting, tailored suggestions to overcome hurdles, and weekly challenges. The VitaBit toolkit supports the participants to monitor their behavior in relation to self-set goals.

**Conclusions:**

Intervention mapping is a useful protocol not only for the systematic development of a comprehensive intervention to reduce sedentary behavior but also for planning program adherence, program implementation, and program maintenance. It facilitates obtaining the participation of relevant stakeholders at different ecological levels in the development process of the intervention and anticipating facilitators to and barriers of program implementation and maintenance.

**Trial Registration:**

Netherlands Trial Register NL7503; https://www.trialregister.nl/trial/7503

## Introduction

### Background

Frequent and uninterrupted sedentary behavior is highly prevalent among office workers [1,2] and negatively impacts workers’ health and well-being by increasing the risk of noncommunicable diseases such as cardiovascular disease, type 2 diabetes [3-5], obesity [6], and mental health problems [7,8]. This is reflected in the higher mortality rates among office workers as compared with those in more active occupations [9]. Sedentary behavior is defined as sitting, lying, or reclining awake behaviors with low-energy expenditures (≤1.5 metabolic equivalents) [10]. Compensating for the negative effects of sitting time by meeting the recommended levels of physical activity may not be possible [11-15]. Moreover, the accumulation of long uninterrupted sitting bouts and/or a daily sitting time of more than 10 hours has been defined as an unhealthy sitting pattern resulting in increased metabolic risk [15,16]. Research suggests that prolonged sitting should be interrupted by bouts of light to moderate physical activity [16,17] and standing [18,19].

Few studies described the long-term positive effects of interventions to reduce sedentary behavior. Interventions mostly incorporated multiple behavior change methods targeting multiple behavioral determinants [20,21]. Behavior change methods are defined as “general techniques or processes that have been shown to be able to change one or more determinants of behavior” and the behavior, if parameters for use are respected [22,23]. For instance, behavior change methods providing information about health consequences and self-monitoring help build the attitude required to decide to change; instructions about how to perform the behavior and social support help build the self-efficacy required to translate the intention into behavior. Establishing a clear link between the identified determinants of behavior and behavior change methods targeting these determinants is a key component of effective behavior change, according to the intervention mapping (IM) protocol [22]. Worksite physical activity interventions designed using IM have revealed positive long-term effects [24-26]. However, current effective sedentary behavior interventions are quite cost-intensive requiring a personal coach and/or environmental changes [26-28]. This paper describes the systematic development of a low-cost data-driven worksite sedentary behavior intervention designed with the IM protocol.

### Intervention Mapping

**Figure 1:**
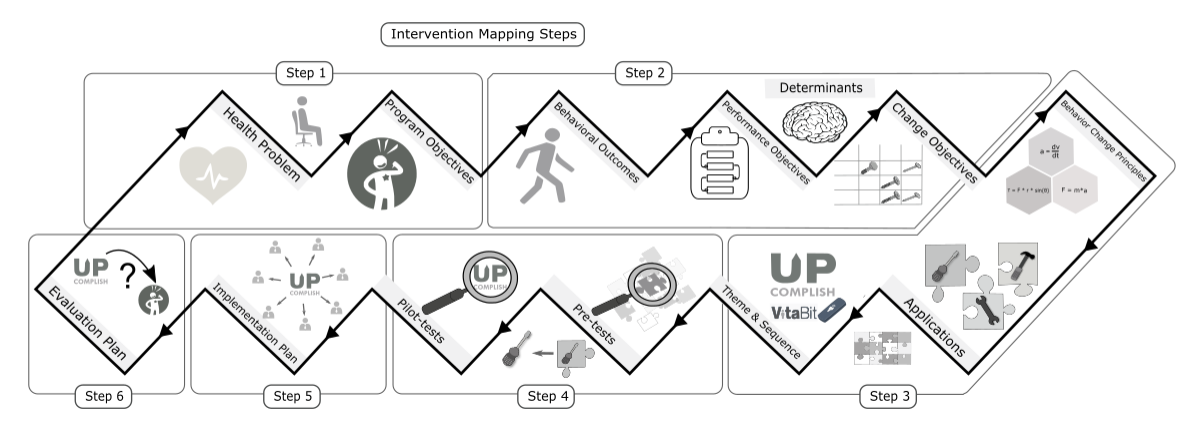
Overview of the steps and products in the intervention mapping protocol.

IM is a framework for planning intervention development, implementation, and evaluation with six iterative steps. In each step, the program designer applies findings from theory, evidence, and their own research: (1) conducting a needs assessment, (2) stating program outcomes and objectives, (3) designing the program, (4) preparing program production, (5) planning program implementation, and (6) developing an evaluation plan (see Figure 1) [22,29].

Sedentary behavior can be embedded at both the interpersonal (ie, support by colleagues and managers) and the individual (ie, office workers) level. For example, if an employee would like to interrupt sitting time more often during working hours but is devaluated by their colleagues for not working enough, the new behavior might disappear. Higher levels (ie, organization, community, and society) were not considered in this study for reasons of cost-effectiveness and given that the target high-income Western countries provide sufficient opportunities (such as safe pathways) for individuals to sit less during working hours.

An intervention planning group includes stakeholders who can make relevant contributions to the development, implementation, and evaluation, such as members of the target group and future implementers. This ensures that issues pertinent to the target group are addressed by the intervention or that future implementation issues are anticipated ahead of time [22].

Computer and smartphone technologies can create platforms that support interactions between individuals, making it possible to exchange both print and more complex multimedia files (eg, a coaching procedure at reduced costs that allows for individually adapted suggestions) [30-32]. Since a permanent reduction of sedentary behavior requires the personal assistance of a professional [33], the main component of our intervention is UPcomplish, which is partly automated, with tailored feedback and motivational support remotely provided by a coach. The VitaBit monitoring toolkit is part of the intervention; participants can monitor their own sedentary behavior related to their personal goals, and the UPcomplish coach can use those data to give almost real-time tailored advice.

In this paper, we describe the systematic development of UPcomplish and the design of the VitaBit monitoring toolkit. IM guided important decisions with regard to objectives, behavior change methods, program production, implementation, and evaluation. The decisions were informed by relevant theoretical and empirical literature including our own empirical research. With UPcomplish and VitaBit, we aim to reduce the number and length of sitting bouts among office workers in the short term [3] and increase the vitality and mental health of employees, as well as minimize their risks for noncommunicable diseases in the longer term.

## Methods

All materials and supporting documents are available at the Open Science Framework (OSF) repository [34]. The target population consists of office workers in high-income countries [35]. The trial was registered with the Netherlands Trial Register [NL7503].

### Intervention Mapping Steps 1 and 2: Needs Assessment and Program Objectives

The first two IM steps cover problem identification and the logic model of change (problem behaviors and desired behaviors, as well as environmental outcomes). The health problem of sedentary behavior, its impact on quality of life, and the context of the intervention were specified (Figure 1). Individual and environmental factors causing sedentary behavior were identified, and behavioral and psychological outcomes stated for the target group (office workers) and the actors at the interpersonal level (colleagues and managers). Behavioral outcomes often comprise more specific subbehaviors (eg, deciding, planning, monitoring), performance objectives, which are influenced by psychosocial determinants (eg, attitude) consisting of subdeterminants (eg, specific beliefs). Only relevant and changeable determinants were identified. Relevance of a determinant refers to the strength of its association with the outcome behavior; changeability refers to the likelihood that the intervention will influence a change in the determinant [22]. We created a matrix, in which performance objectives constitute the rows, and the relevant and changeable determinants the columns. The cells represent the change objectives and provide detailed and measurable information on who and what will change, providing the basis of our intervention.

### Intervention Mapping Steps 3 and 4: Program Design and Production

During IM step 3, we selected behavior change methods based on their suitability to cause change in the determinants that needed to be targeted. These were then translated into practical applications by matching the methods to change objectives considering the parameters of use. We focused on a tailored intervention based on two components (each with several objectives), the VitaBit measurement toolkit and the content of UPcomplish (supplied by the personal coach). We further specified scope and sequence of the program and the program theme. In IM step 4, the practical applications were arranged into a coherent program. Program messages and intervention components were drafted and pilot-tested before being refined and produced.

### Intervention Mapping Step 5: Adoption and Implementation Plan

In IM step 5, an adoption, implementation, and sustainability plan was created to maximize the likelihood of maintaining behavioral effects and address program dissemination, structural implementation, and maintenance of the intervention. Relevant stakeholders were identified. Behavioral outcomes were formulated and linked to important determinants. The resulting change objectives were used to map an intervention for adopters, implementers, and maintainers by reapplying IM steps 3 and 4.

### Intervention Mapping Step 6: Evaluation Plan

IM step 6 focuses on planning an evaluation to determine behavioral and health effects and underlying mechanisms of intervention effectiveness. We collected and designed indicators and measures and planned the design and procedure of the evaluation study.

## Results

### Intervention Mapping Steps 1 and 2: Needs Assessment and Program Objectives

#### Program Objectives

Different sedentary behavior parameters have been recommended [36]. This lack of consensus is rooted in both differences in predicted health outcomes (ie, coronary heart diseases vs type 2 diabetes) and recommended behavioral outcomes (ie, daily sitting time vs daily amount of light activity). As a behavioral outcome regarding sedentary behavior, we considered the recommended values from three cohort studies investigating diseases relevant to the target group (ie, heart diseases, diabetes, and all-cause mortality) [9,14,37]. The program objective includes three subobjectives: reduction in daily sitting time, increase in daily light activity, and attainment of a healthy sitting pattern (including fewer long and uninterrupted sitting bouts). The first two subobjectives were set at a daily sitting time of less than 8 hours per day per person [9,14,37] and a minimum of 4 hours standing and light activity per day [36].

**Figure 2:**
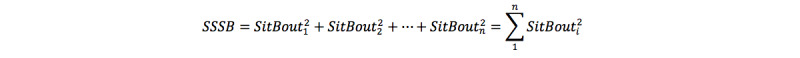
Equation summed squared sitting bouts.

While not only total sitting time is important but also regular sitting interruptions, there is no direct empirical support for the recommendation of a particular sitting pattern. In order to represent the daily sitting pattern, we propose to square the lengths of the daily sitting bouts and to sum them up (summed squared sitting bouts [SSSB]) as shown in Figure 2.

**Figure 3:**
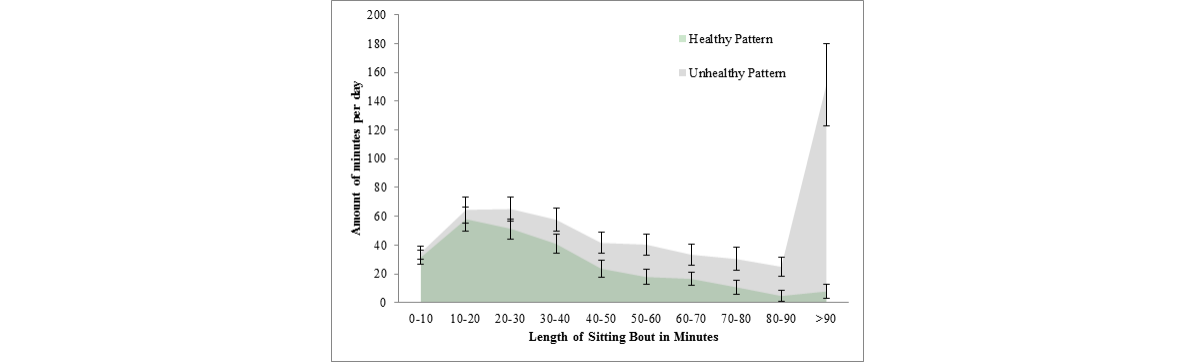
Healthy versus unhealthy summed squared sitting bouts days in the pilot study.

As this is a new representation, a cutoff recommendation relating this value to health outcomes has not yet been investigated. Therefore, based on our baseline activity data (n=69, see OSF repository), we distinguished between healthy and unhealthy sitting patterns by using the median across all days of SSSB as the cutoff (18.8 * 10^3^ min²). We used the median because the first two subobjectives (sitting and light activity time) were met on about 50% of the days. However, this still needs to be investigated with health outcomes. In spite of similar daily absolute and relative sitting times (see OSF repository), the average duration of sitting bouts collected in longer sitting bouts is significantly smaller on healthy SSSB days, while the amount of sitting in shorter bouts seems to be similar (Figure 3). An SSSB below 18.8 * 10^3^ min² will constitute a healthy sitting pattern according to this pilot study.

Figure 3 represents different average daily sitting minutes collected in certain bout durations on healthy and unhealthy SSSB days (below and above 18.8 * 10³ min²) in the pilot study. The longer the sitting bout, the less it is represented in a healthy pattern, while time spent in very short sitting bouts is similar between healthy and unhealthy SSSB days. For example, on healthy SSSB days, the individuals spent on average 7.8 minutes of the day in long sitting bouts over 90 minutes (including days without any of these long bouts), while on unhealthy SSSB days, the average time spent in those long bouts was 151.4 minutes. The areas under the curve, therefore, represent the averages of total daily sitting time. Although the average overall sitting time does not differ significantly between healthy and unhealthy SSSB days, this graph clearly shows that on a healthy SSSB day, fewer minutes were collected in longer sitting bouts. We assume that the two sitting patterns differ in terms of health outcome.

The participants in our pilot study met the sitting time objective (maximum 8 hours) with an average of 3.1 days (58.8% of their wearing days), the standing and light activity time objective (minimum 4 hours) with an average of 3.3 days (50.9% of their wearing days), and the SSSB objective (maximum 18.8 * 10^3^ min²) with an average of 2.9 days (54.0% of their wearing days). All three subobjectives were met on an average of 1.4 days (22.8%). Consequently, we specified the following program goal: Participants should achieve all three recommendations on at least 30% of the wearing days in a week (including weekend days). This, at the baseline measurement, was achieved by 26.1% of the participants (control event rate [38]). We would therefore determine effectiveness by the difference of the proportion of participants who meet the program goal after receiving the intervention compared with baseline.

#### Behavioral Outcomes and Performance Objectives for the Individual Office Worker

At the individual level, the behavioral outcomes were split into a preintentional motivational phase, building an intention to reduce sedentary behavior and preparing for change, and a postintentional volitional phase, translating the intention into behavior [39]. The first behavioral outcome: employees launch a self-regulatory process of controlling their sedentary behavior. This starts with questioning the current behavior and forming an intention to change. It includes monitoring behavior and ends with concrete action planning as indicated by self-set goals. The second behavioral outcome: employees engage in activities in accordance with their previously formulated goals. This focuses on the translation of intentions into behavior by overcoming barriers and actual regular interruptions of sedentary behavior. In addition to this self-regulatory process, other desired behavioral outcomes of the program include establishing good habits and preparing participants for relapses [26,40].

#### Behavioral Outcomes and Performance Objectives at the Interpersonal Level

At the interpersonal level, support by colleagues and supervisors is important [30]. Approval from both stakeholders therefore needs to be encouraged and clearly demonstrated. After colleagues and supervisors have decided to show their support, they can apply different supporting strategies. They could decide to participate in a challenge sharing effective strategies for reducing sitting time, as well as joining in and/or initiate standing or walking meetings [41,42]. The support of the supervisors and managers is additionally reflected in the allocation of a room for the kick-off meeting and provision of the funding for the intervention. More information about these two behavioral outcomes can be found in the adoption plan in IM step 5. Supervisors and managers can participate in the program themselves providing similar support to that of the colleagues of the target group [43].

#### Determinants and Change Objectives at the Individual and Environmental Levels

Empirical evidence from previous sedentary behavior studies was garnered to discover determinants for each performance objective. Since standing is often perceived as being more exhausting than sitting, we included evidence from physical activity research [26]. Identified determinants and their synonyms were covered by the reasoned action approach [44] and the extended parallel process model [41]. The temporal self-regulation theory for physical activity [45] was considered to facilitate the translation of intentions into actual behaviors.

Attitudes, perceived social norms, and perceived behavioral control have been shown to explain about 33% of the variance of intention to be less sedentary at work, while 37% of the variance of actual sedentary behavior at work is explained through intention [46]. Since the act of providing support (at an interpersonal level) is a reasoned action, those determinants were also used for agents at the interpersonal level. At the individual level, perceived susceptibility was added as a determinant. A person might only consider making a change if they feel that the threat of negative health outcomes from too much sitting is likely to impact them [47].

**Figure 4:**
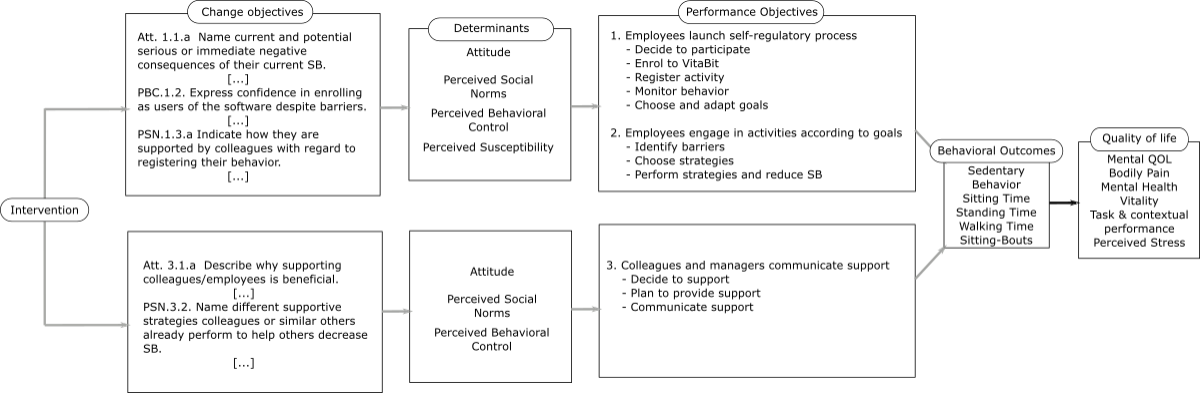
Illustration of the logic model of change.

Specific underlying beliefs were used to develop change objectives, informed by qualitative literature [26,48] and focus group interviews. For example, in order for an individual to participate, the perceived need to be more active (attitude) and the outlook to receive support (injunctive norm) are critical [49]. The concerning change objective: employees name current and potential serious or immediate negative consequences of their current sedentary behavior. From the temporal self-regulation theory for physical activity, the change objectives related to attitude included the importance of the perceived benefits as being greater and sooner, while the perceived costs were smaller and later. Making those benefits and costs salient at choice time was addressed by the change objectives listed under perceived susceptibility [45]. All change objectives are displayed in the matrices of change objectives (see OSF repository for the matrices and the complete logic model of change). Figure 4 illustrates the logic model of change.

### Intervention Mapping Steps 3 and 4: Program Design and Production

#### Behavior Change Methods and Practical Applications

**Figure 5:**
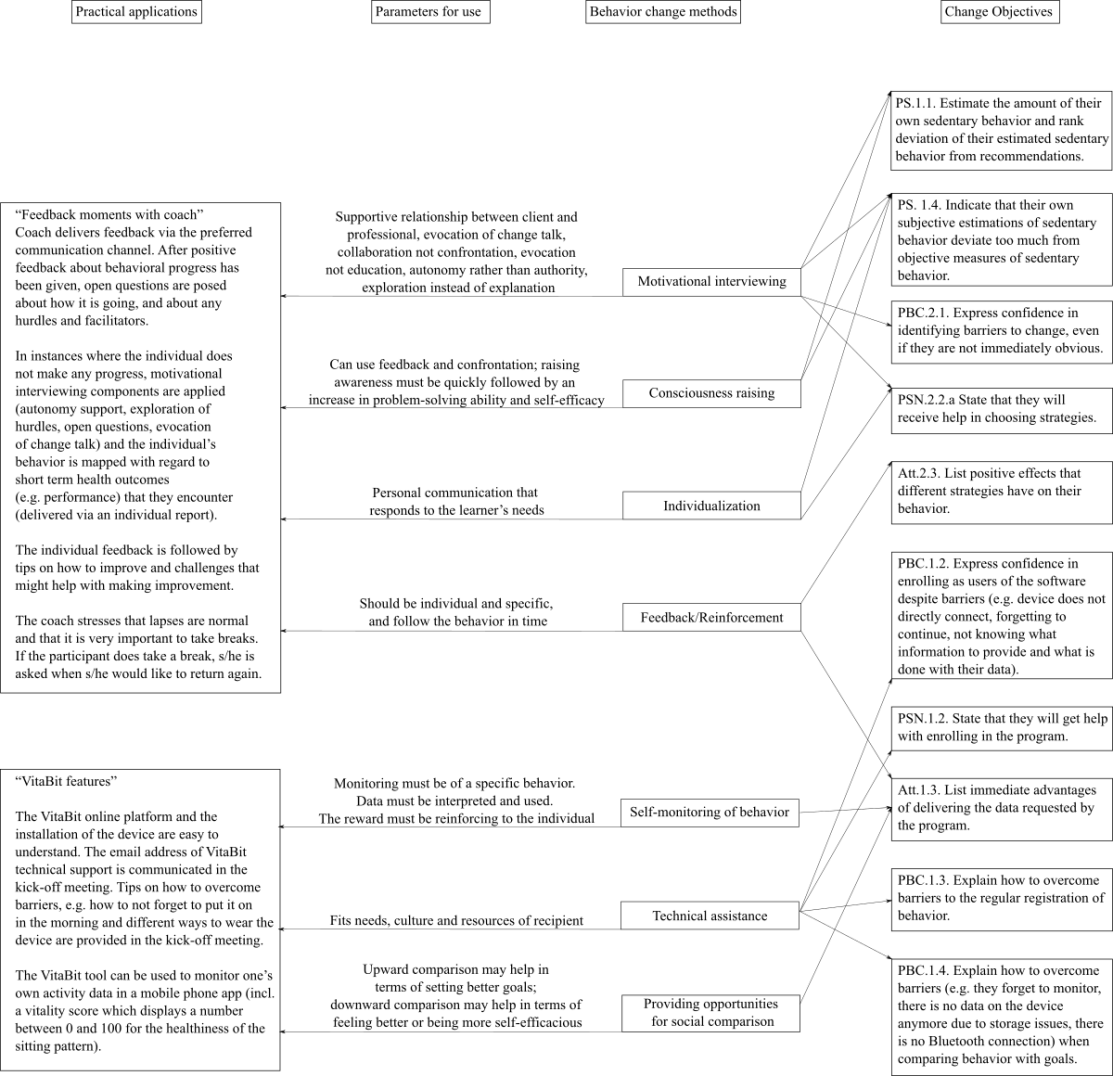
Examples of practical applications.

VitaBit provides the basis for monitoring and delivering individual data, while UPcomplish is provided by a coach to help participants improve their sitting pattern by overcoming individual hurdles. Health professionals and vitality coaches from the field will be the implementers of the intervention, using partly automatized components of UPcomplish (IM step 5). The practical applications can be found in the acyclic behavior change diagrams in the OSF directory, and Figure 5 illustrates examples of important practical applications.

#### Program Theme and Sequence

The theme of UPcomplish is based on the assumption that behavioral change in a professional setting should not be too invasive but still motivational. Therefore, the main factors are challenge and low invasiveness. UPcomplish consists of the word up, indicating the goal of the program is supporting desk workers to stand up, and the word accomplish, which reflects the challenging character of the intervention. Getting UP will be accomplished.

The initial phase of preparation and kick-off provides the foundation for the relationship between participant and coach. Participants are introduced to the VitaBit toolkit, familiarize themselves with their own behavior, and get to know the coach. During the kick-off meeting, individualized goals are set, the importance of interrupting sitting is explained, and the preferred communication channel between coach and participant is agreed upon. The baseline phase continues with behavioral and vitality measurements; participants use the VitaBit device for at least 1 week and complete vitality, health, and performance questionnaires including the task and contextual performance subscale of the Individual Work Performance Questionnaire, the Perceived Stress Scale, and the bodily pain, mental health and vitality subscales of the 36-item Short Form Health Survey [50-53] (first of 3 times). During the 3-month trajectory with the coach, participants are provided with activity challenges in biweekly circles. They receive feedback about their behavior 2 times per week and discover facilitators of and hurdles to their behavior through motivational interviewing components. Goals are adjusted after 4 and 8 weeks. In the middle of the intervention, after 6 weeks, participants complete the vitality questionnaire for the second time. In the last 2 weeks, there is a focus on building up habits supported by implementation intentions and the use of buddy systems. At this stage, the vitality questionnaire is completed for the last time [45,54,55]. A group report and individual vitality feedback provide an overview of the participant’s achievements (see OSF directory).

#### Pretests of Program Materials

In order to determine whether the program can be implemented, it needs to be pretested and pilot tested. Pretesting refers to the process whereby specific components of the intervention are tested among the intended population before final production. The goal of pretesting is to safeguard the conditions for effectiveness of the behavior change methods in each component.

Pilot testing is the last evaluation involving all program components, the intended population, and implementers prior to the actual implementation. The goal is to assess the acceptability of the entire program and anticipate any problems in implementation [22].

#### VitaBit Monitoring Toolkit Pretest

The VitaBit toolkit consists of an accelerometer, mobile phone app, and complementary online platform. These provide the user with tools to monitor their posture patterns with the help of a vitality score (0 = unhealthy, 100 = healthy), set short- and long-term goals, and compare their performance with that of other users. The VitaBit device is a small (3.9 × 1.4 × 0.85 cm, 4.8 g) triaxial wearable accelerometer that monitors sitting, standing, and activity behavior on a half-minute-by-half-minute basis. With regard to sitting, it shows sensitivity and specificity values of 85.7% and 91.2%, respectively [56].

Before the release of the VitaBit toolkit, over 50 pretesters (exact number was not documented) from potential organizations were allocated the VitaBit device, asked to use the device for as long as they liked, and later contacted to provide feedback. This feedback provided information about functionality, design, and features and was translated into improving software components by the VitaBit development team.

#### UPcomplish Pretest

Initial UPcomplish components were pretested in 11 dispatchers from a German control center. Standing desks were available to these individuals, whose duties mainly involved desk work. A kick-off meeting entailed discussions about the importance of being less sedentary and a short explanation about the intervention and its development. Participants received a weekly progress report. Individual hurdles and facilitators were discussed via their preferred communication channel. Each week, participants received a message in which different performance objectives were addressed, depending on former behaviors and/or reactions to messages (ie, week 1: monitoring behavior; week 2: goal setting; week 3: identifying barriers; etc; see OSF directory). Challenges and other aspects of gamification were not yet included. Two focus group discussions and individual phone calls with participants of this pretest provided feedback about the intervention suggesting that the videos were not watched because they were perceived as being too long, too difficult to download, or too difficult to understand. These video clips were therefore removed from UPcomplish. The kick-off created an atmosphere of trust. However, due to the information about the intervention development being perceived as too lengthy, we decided to shorten the session. The kick-off meeting was also used to help the participants who had not yet tried or succeeded in connecting their device. We decided to split these program components up and call them account creation and pairing the device and that account creation should already be covered before future kick-off meetings to avoid some participants having to wait around. Pairing the device should be handled after the kick-off meeting, in case participants want to directly pair their device with support. The inclusion of challenges and aspects of gamification were not included in this pretest. However, we assumed that these would be attractive and helpful elements. In addition to tailored psychological advice, tailored health advice on individual health outcomes was perceived to be potentially helpful. We decided that motivational interviewing questions should be shortened and performance objectives addressed more frequently, resulting in more frequent delivery of more concise information. Participants showed interest in the vitality score, which provided them with a value between 0 and 100 of how healthy their sitting pattern was.

#### Pilot Test of UPcomplish

After all adaptations had been made, based on the results of our pretesting, 23 public service desk workers from the Netherlands (5 in the UPcomplish group, who explicitly asked to receive the intervention) took part in our pilot test. After the kick-off meeting, each participant in the UPcomplish group received feedback 2 times per week via their preferred communication channel: individual feedback about goal achievement over the previous week and information regarding sitting patterns on certain weekdays. Furthermore, facilitators of and barriers to sitting less were discussed. Every 2 weeks, participants received gamified challenges. After 4 and 8 weeks, individual goals were revised, if necessary. Summarizing reports completed the intervention. All participants of UPcomplish remained in the program until the end and perceived the coaching to be helpful in terms of reducing sitting time. On average per week, they wore the VitaBit on 74.6% of the days. We observed improvements of sitting, standing, and activity time but cannot interpret them due to the low number of participants and the selectivity of the sample.

### Intervention Mapping Step 5: Adoption and Implementation Plan

We expect the managers of our target companies to adopt the intervention, as indicated by the provision of financial funding for the intervention and provision of a room for the kick-off meeting. Additionally, they will supervise and oversee the sustainability of the intervention and its effects. In order to adopt the intervention, the managers first should identify a need to make a decision (eg, determinant: attitude). Second, they should prioritize UPcomplish for individual reasons, such as for an improved reputation of the company (eg, determinant: attitude). Eventually, they should subscribe to the program and continue the subscription for the long run (eg, determinant: perceived behavioral control and attitude) while supervising behavioral maintenance of their employees or institutionalizing the program [43].

Personal talks with the management will address diverse underlying buying preferences. A regular report linking average activity and rates of dropout and commitment, among others, to short-term effects on vitality, performance, mental health, and perceived stress will facilitate positive outcome expectancies. A separate study linking the health outcomes to return on investment is in the planning.

Health professionals and vitality coaches from the field are the implementers of this intervention. It is essential that every component is delivered in the suggested tailored and supportive way in order to maintain program fidelity. Completeness will be accomplished if users receive all of the program components. A workshop for data-driven coaching and meetings with the coaches that implement UPcomplish will maximize fidelity and completeness. A coaching portal in the VitaBit dashboard helps the coach to easily supervise their participants by getting an overview of individual activity patterns. Buttons next to the values of the participants make it possible to deliver the coaches’ suggestions directly to the relevant participants or to get an overview of their dashboards. Multimedia Appendix 1 shows an example of the coaching portal. The average sitting, standing, and walking parameters for a given period of time are displayed on one page. On the right, the coach can send individual notifications and emails, inspect the individual portal to get more detailed insights into the daily activity behaviors, and create new widgets, such as setting a new goal.

Mobile phone–based workplace sedentary behavior interventions seem to be especially effective in the medium term (3- to 6-month follow-up) if they incorporate several behavior change methods [21]. These include self-monitoring and prompts or cues combined with information about health consequences and information about how to perform the desired behavior. In order to facilitate program sustainability, it is important to tailor the maintenance intervention to the participants who sit the most during their workdays, or, more generally, to those with different motivational profiles, such as a focus on health promotion versus weight loss versus illness prevention [21,57]. The coaches are encouraged to stress the importance of buddy systems and deliver regular short and precise health information in order to stabilize attitudes about sedentary behavior in the target group. Optional email reminders and health blogs help users to be reminded of the importance of reducing sitting time. Analyzing dropouts in the process evaluation and preventing reasons for future dropouts will help to facilitate program sustainability [22].

### Intervention Mapping Step 6: Evaluation Plan

We plan to evaluate short-term effectiveness in terms of decreased sitting time, SSSBs, and increased standing and walking time (secondary effects on short-term quality-of-life outcomes [7,58,59]) of UPcomplish (effect evaluation). Furthermore, we will consider whether any program adaptations are needed and what these might be (process evaluations). We will employ a multilevel design with between-subjects and within-subjects factor (measurement moment) comparisons and estimate the intervention’s effect in a magnitude of standard deviations (Cohen *d*) to enable the computation of the number needed to treat (number of people that should receive the intervention for one person to change their behavior sufficiently to meet the criteria specified in the intervention goal [38]). The number needed to treat can be used to calculate the cost of the intervention needed for at least 30% of the participants to achieve all three behavioral outcomes.

From May 2019 until January 2020, we had 200 VitaBit monitors at our disposal. We chose a stepped-wedge design (last week of one group is compared with first week of another group) because a control group (VitaBit only) was not possible considering high expected dropout rates and feasibility issues. Splitting up intervention groups into as many groups as possible would reveal a bigger sample size since some groups could provide data for both the baseline and postintervention measurement. Having five different intervention groups was considered the minimum yet doable number of groups where one group can provide data for the two measurements. The five intervention groups, each comprising 40 participants, start with a time lag of 7 weeks. With an anticipated retention rate of 80%, this yields an analyzable sample size of n=192 [60]. With 192 participants, estimation of this effect size is accurate to about a quarter of the standard deviation (see the OSF repository for details and a flowchart illustrating the design).

The process evaluation is informed by qualitative and quantitative output from surveys and behavioral data and will assess both important aspects of the logic model of change and intervention components.

#### Procedure

Groups of 10 to 15 desk workers from random companies in Germany are recruited via email and personal contacts. Potential groups are randomly assigned to one of the intervention groups and informed about the intervention and the measurements before consent is obtained. Each group receives the 12-week intervention and is requested to complete vitality, performance, and mental health questionnaires at 3 points in time. Participants can refuse participation in the intervention and/or the measurements at any times without giving a reason. The evaluation of this intervention including its consent procedure was approved by the Ethical Review Committee, Psychology and Neuroscience, Maastricht University, the Netherlands (ERCPN- 188_11_02_2018). More details can be found in “The Evaluation of UPcomplish: Sample size planning and procedure” in the OSF directory.

#### Measures Process Evaluation

All questionnaires can be found in the OSF repository and were translated into German using the back-translation method if no validated German version was available [61]. Table 1 provides an overview of all measurements that are used in the evaluation.

**Table 1:** Measurements and example items.

Variable		Measurements and indicators	Items	Example item	Point in time
**Intervention characteristics**					
	Acceptability	Taken from a former evaluation [48]	18	“The questions within the recommendations were clear.”	End
	Fidelity	Messages from automated pool divided by total amount of messages sent by the coach	N/A^a^	N/A	End
	Reach	Dropout rate; ratio of participants from the intended target group; dose received	N/A	N/A	End
**Determinants**					
	Attitude	Taken from a former evaluation [48]	6	“Standing and walking around at work is healthy.”	Baseline, middle, end
	PSN^b^	Taken from a former evaluation [48]	2	“Standing and walking around at work is encouraged by my colleagues.”	Baseline, middle, end
	PBC^c^	Taken from a former evaluation [48]	4	“I am sure that I can stand and walk around at work, even though I feel bad, tired, tense or depressed.”	Baseline, middle, end
	Perceived susceptibility	Self-created questions to assess perceived susceptibility to improper sitting habits	2	“My daily sitting time is more than what is recommended.”	Baseline, middle, end
**Performance objectives**					
	PO^d^ 1.2 Enrollment as VitaBit user	Proportion of successfully enrolled participants among the ones who agreed to participate	N/A	N/A	End
	PO 1.3 Registration of sedentary and antagonistic behaviors	Average of days per week that show VitaBit data for at least 6 hours	N/A	N/A	End
	PO 1.4 Monitoring of behavior	Number of days missing before the feedback moments	N/A	N/A	End
	Action planning, identifying barriers and facilitators, and support	Numbers and quality of responses to coaching questions/requests	N/A	N/A	End
**Sedentary behavior and physical activity**					
	Objectively measured sitting (30-second periods)	VitaBit measurement toolkit [56,62]	N/A	N/A	continuously
	Moderate and vigorous physical activity	German version of the International Physical Activity Questionnaire (short form) [63]	max. 6	“During the last 7 days, on how many days did you do vigorous physical activities like heavy lifting, digging, heavy construction, or climbing stairs as part of your work? Think about only those physical activities that you did for at least 10 minutes at a time.”	Baseline, middle, end
**Secondary outcome: quality-of-life**					
	Task and contextual performance	Two subscales of the Individual Work Performance Questionnaire [50]	14	“In the past week, I took on extra responsibilities.”	Baseline, middle, end
	Stress perception	Perceived Stress Scale [51,53]	10	“In the last week, how often have you felt nervous and “‘stressed’?”	Baseline, middle, end
	Bodily pain	Subscale of the SF^e^-36 health survey [52]	2	“How much bodily pain have you had during the past week?”	Baseline, middle, end
	Mental health	Subscale of the SF-36 health survey [52]	5	“How much of the time during the past week have you been a happy person?”	Baseline, middle, end
	Vitality	Subscale of the SF-36 health survey [52]	4	“How much of the time during the past week did you have a lot of energy?”	Baseline, middle, end
**Covariates: demographic, educational, and job-related variables**					
	Gender, age, educational level, height, weight, and job-related variables (eg, team size)	Measured by VitaBit during account creation	8	N/A	Baseline
	Job tasks	Taken from a former evaluation [48]	5	“How much on average per day (in %) do you estimate you spend on the following tasks? Phone calls?”	Baseline
	Employment status and working times	Self-created questions	2	“How many days do you usually work in a week?”	Baseline

^a^N/A: not applicable.

^b^PSN: perceived social norms.

^c^PBC: perceived behavioral control.

^d^PO: performance objective.

^e^SF: Short Form Health Survey.

### Statistical Analyses

Statistical analyses encompass multilevel analyses. For the between-subject comparisons, the outcome variables are centered around baseline company means, and the analyses are nested by calendar week. For the within-subject comparisons, the outcome variables are centered around calendar weeks, and the analyses are nested on the individual level. Analyses are adjusted for possible confounding variables such as company-related variables, gender, or age.

Multilevel linear and logistic regressions are conducted to inspect putative effects of performance objectives and determinants on the continuous primary outcome variables and the dichotomous performance objectives (performed yes/no), respectively.

## Discussion

### Principal Findings

This paper describes an IM protocol to develop a computer-based intervention aimed at reducing sedentary behavior at work. A tailored intervention was developed to guide participants step by step through a behavioral change process. The support of both colleagues and supervisors was considered and addressed in additional components. A plan to ensure adoption, implementation, and sustainability was drafted. Finally, we developed an evaluation plan for assessing the effects of the intervention and the mechanisms behind these effects.

### Strengths and Limitations

Although the IM approach suggests working through all the core processes, not all substeps were performed in our study [64], partly due to the fact that additional research (eg, about the necessity of all behavioral substeps [ie, performance objectives]) was still ongoing. Still, we plan to complete a process evaluation that will investigate mechanisms of effectiveness and provide additional information. A second limitation is that members of the target group and managers of companies who might potentially use the intervention (except those working at VitaBit) were contacted too late to be part of the planning group since they were only contacted as part of the pretest and pilot test. Nevertheless, the interest in reducing sedentary behavior seems to be high, and multiple informal talks during the development process with potential adopters, implementers, and people from the target group have revealed valuable insights.

A benefit of the project was the collaboration between scientific research and information technology practice. To facilitate this collaboration, face-to-face and Skype discussions were used to directly exchange ideas and possibilities. In doing so, we also discovered more challenging aspects of collaboration between health promotion and information technology practice. The usage of technical terms on both sides, different priorities during the development process, and balancing act between tailoring and standardization are examples of the challenges we encountered. However, working together allowed for a quick translation of knowledge about behavioral change into practical applications and provides an example that can be applied to other IM procedures [65,66].

### Conclusion

We developed a comprehensive intervention targeting important determinants at two different ecological levels. The development of our intervention was grounded in relevant literature, and multiple theories have been applied. Future evaluation studies should investigate the program effectiveness and further analyze the relevance and utility of single program components.

## Appendix

Multimedia Appendix 1 Coaching overview of VitaBit portal.

## Abbreviations

IM: intervention mapping
OSF: Open Science Framework
SSSB: summed squared sitting bouts

## References

[1] Ryan CG, Dall PM, Granat MH, Grant PM (2011). Sitting patterns at work: objective measurement of adherence to current recommendations. Ergonomics.

[2] Clemes SA, O'Connell SE, Edwardson CL (2014). Office workers' objectively measured sedentary behavior and physical activity during and outside working hours. J Occup Environ Med.

[3] Biswas A, Oh PI, Faulkner GE, Bajaj RR, Silver MA, Mitchell MS, Alter DA (2015). Sedentary time and its association with risk for disease incidence, mortality, and hospitalization in adults: a systematic review and meta-analysis. Ann Intern Med.

[4] Wilmot EG, Edwardson CL, Achana FA, Davies MJ, Gorely T, Gray LJ, Khunti K, Yates T, Biddle SJH (2012). Sedentary time in adults and the association with diabetes, cardiovascular disease and death: systematic review and meta-analysis. Diabetologia.

[5] van Uffelen JGZ, Wong J, Chau JY, Riphagen I, Gilson ND, Burton NW, Healy GN, Thorp AA, Clark BK, Gardiner PA, Dunstan DW, Bauman A, Owen N, Brown WJ (2010). Occupational sitting and health risks: a systematic review. Am J Prev Med.

[6] Chau JY, Merom D, Chey T, Bauman AE (2012). Cross-sectional associations between occupational and leisure-time sitting, physical activity and obesity in working adults. Prev Med.

[7] Hamer M, Stamatakis E (2014). Prospective study of sedentary behavior, risk of depression, and cognitive impairment. Med Sci Sports Exerc.

[8] Voss M, Carr L, Clark R, Weng T (2014). Revenge of the “sit” II: Does lifestyle impact neuronal and cognitive health through distinct mechanisms associated with sedentary behavior and physical activity?. Mental Health Phys Activ.

[9] Chau JY, Grunseit A, Midthjell K, Holmen J, Holmen TL, Bauman AE, Van der Ploeg HP (2015). Sedentary behaviour and risk of mortality from all-causes and cardiometabolic diseases in adults: evidence from the HUNT3 population cohort. Br J Sports Med.

[10] Tremblay MS, Aubert S, Barnes JD, Saunders TJ, Carson V, Latimer-Cheung AE, Chastin SFM, Altenburg TM, Chinapaw MJM, SBRN Terminology Consensus Project Participants (2017). Sedentary Behavior Research Network (SBRN) - terminology consensus project process and outcome. Int J Behav Nutr Phys Act.

[11] Hamilton MT, Healy GN, Dunstan DW, Zderic TW, Owen N (2008). Too little exercise and too much sitting: inactivity physiology and the need for new recommendations on sedentary behavior. Curr Cardiovasc Risk Rep.

[12] Thorp AA, Owen N, Neuhaus M, Dunstan DW (2011). Sedentary behaviors and subsequent health outcomes in adults a systematic review of longitudinal studies, 1996-2011. Am J Prev Med.

[13] Ekelund U, Steene-Johannessen J, Brown WJ, Fagerland MW, Owen N, Powell KE, Bauman A, Lee I, Lancet Physical Activity Series 2 Executive Committe, Lancet Sedentary Behaviour Working Group (2016). Does physical activity attenuate, or even eliminate, the detrimental association of sitting time with mortality? A harmonised meta-analysis of data from more than 1 million men and women. Lancet.

[14] Pandey A, Salahuddin U, Garg S, Ayers C, Kulinski J, Anand V, Mayo H, Kumbhani DJ, de Lemos J, Berry JD (2016). Continuous dose-response association between sedentary time and risk for cardiovascular disease: a meta-analysis. JAMA Cardiol.

[15] Bankoski A, Harris TB, McClain JJ, Brychta RJ, Caserotti P, Chen KY, Berrigan D, Troiano RP, Koster A (2011). Sedentary activity associated with metabolic syndrome independent of physical activity. Diabetes Care.

[16] Healy GN, Dunstan DW, Salmon J, Cerin E, Shaw JE, Zimmet PZ, Owen N (2008). Breaks in sedentary time: beneficial associations with metabolic risk. Diabetes Care.

[17] Dunstan DW, Kingwell BA, Larsen R, Healy GN, Cerin E, Hamilton MT, Shaw JE, Bertovic DA, Zimmet PZ, Salmon J, Owen N (2012). Breaking up prolonged sitting reduces postprandial glucose and insulin responses. Diabetes Care.

[18] Owen N, Sugiyama T, Eakin EE, Gardiner PA, Tremblay MS, Sallis JF (2011). Adults' sedentary behavior determinants and interventions. Am J Prev Med.

[19] Gupta N, Heiden M, Aadahl M, Korshøj M, Jørgensen MB, Holtermann A (2016). What Is the effect on obesity indicators from replacing prolonged sedentary time with brief sedentary bouts, standing and different types of physical activity during working days? A cross-sectional accelerometer-based study among blue-collar workers. PLoS One.

[20] Gardner B, Smith L, Lorencatto F, Hamer M, Biddle SJ (2015). How to reduce sitting time? A review of behaviour change strategies used in sedentary behaviour reduction interventions among adults. Health Psychol Rev.

[21] Stephenson A, McDonough SM, Murphy MH, Nugent CD, Mair JL (2017). Using computer, mobile and wearable technology enhanced interventions to reduce sedentary behaviour: a systematic review and meta-analysis. Int J Behav Nutr Phys Act.

[22] Bartholomew Eldredge LK, Markham CM, Ruiter RA, Fernández ME, Kok G, Parcel GS (2016). Planning Health Promotion Programs: An Intervention Mapping Approach, 4th Edition.

[23] Kok G, Gottlieb NH, Peters GY, Mullen PD, Parcel GS, Ruiter RAC, Fernández ME, Markham C, Bartholomew LK (2015). A taxonomy of behaviour change methods: an Intervention Mapping approach. Health Psychol Rev.

[24] Coffeng JK, Boot CRL, Duijts SFA, Twisk JWR, Hendriksen IJM (2014). Effectiveness of a worksite social & physical environment intervention on need for recovery, physical activity and relaxation; results of a randomized controlled trial. PLoS One.

[25] Kwak L, Kremers SP, Candel MJ, Visscher TL, Brug J (2010). Changes in skinfold thickness and waist circumference after 12 and 24 months resulting from the NHF-NRG In Balance-project. Int J Behav Nutr Phys Act.

[26] McEachan RRC, Lawton RJ, Jackson C, Conner M, Lunt J (2008). Evidence, theory and context: using intervention mapping to develop a worksite physical activity intervention. BMC Public Health.

[27] Coffeng JK, Hendriksen IJM, Duijts SF, Proper KI, Boot CRL (2012). The development of the Be Active & Relax. BMC Public Health.

[28] Kwak L, Kremers SPJ, Werkman A, Visscher TLS, van Baak MA, Brug J (2007). The NHF-NRG in balance-project: the application of intervention mapping in the development, implementation and evaluation of weight gain prevention at the worksite. Obes Rev.

[29] Crutzen R, Peters GY (2018). Evolutionary learning processes as the foundation for behaviour change. Health Psychol Rev.

[30] Schoeppe S, Alley S, Bray NA, Williams SL, Duncan MJ, Vandelanotte C (2016). Efficacy of interventions that use apps to improve diet, physical activity and sedentary behaviour: a systematic review. Int J Behav Nutr Phys Act.

[31] Broekhuizen K, Kroeze W, Oenema A, Brug J (2012). A systematic review of randomized controlled trials on the effectiveness of computer-tailored physical activity and dietary behavior promotion programs: an update. Ann Behav Med.

[32] Kelders SM, Kok RN, Ossebaard HC (2012). Persuasive system design does matter: a systematic review of adherence to web-based interventions. J Med Internet Res.

[33] Gardner B, Smith L, Lorencatto F, Hamer M, Biddle SJ (2015). How to reduce sitting time? A review of behaviour change strategies used in sedentary behaviour reduction interventions among adults. Health Psychol Rev.

[34] Preventing sedentary behavior at work: An Intervention Mapping approach for data-driven mhealth consulting. Open Science Framework repository.

[35] Guthold R, Stevens GA, Riley LM, Bull FC (2018). Worldwide trends in insufficient physical activity from 2001 to 2016: a pooled analysis of 358 population-based surveys with 1·9 million participants. Lancet Glob Health.

[36] Buckley JP, Hedge A, Yates T, Copeland RJ, Loosemore M, Hamer M, Bradley G, Dunstan DW (2015). The sedentary office: an expert statement on the growing case for change towards better health and productivity. Br J Sports Med.

[37] van der Berg JD, Stehouwer CDA, Bosma H, Willems PJB, Savelberg HHCM, Schram MT, Sep SJS, Henry RMA, Dagnelie PC, Schaper NC, Koster A (2016). Associations of total amount and patterns of sedentary behaviour with type 2 diabetes and the metabolic syndrome: The Maastricht Study. Diabetologia.

[38] Gruijters S, Peters GJ (2017). Gauging the impact of behavior change interventions: a tutorial on the numbers needed to treat. PsyArXiv.

[39] Schwarzer R (2008). Modeling health behavior change: how to predict and modify the adoption and maintenance of health behaviors. Appl Psychol.

[40] Sniehotta FF, Presseau J, Hobbs N, Araújo-Soares V (2012). Testing self-regulation interventions to increase walking using factorial randomized N-of-1 trials. Health Psychol.

[41] Ball K, Bauman A, Leslie E, Owen N (2001). Perceived environmental aesthetics and convenience and company are associated with walking for exercise among Australian adults. Prev Med.

[42] Giles-Corti B, Donovan RJ (2003). Relative influences of individual, social environmental, and physical environmental correlates of walking. Am J Public Health.

[43] Butterfoss F, Kegler M, Francisco V, Glanz K, Rimer B, Viswanath K (2008). Mobilizing organizations for health promotion: theories of organizational change. Health Behavior and Health Education: Theory, Research, and Practice, 4th Edition.

[44] Fishbein M (2011). Predicting and Changing Behavior: The Reasoned Action Approach.

[45] Hall PA, Fong GT (2015). Temporal self-regulation theory: a neurobiologically informed model for physical activity behavior. Front Hum Neurosci.

[46] Prapavessis H, Gaston A, DeJesus S (2015). The Theory of Planned Behavior as a model for understanding sedentary behavior. Psychol Sport Exercise.

[47] Witte K (1992). Putting the fear back into fear appeals: the extended parallel process model. Comm Monographs.

[48] De Cocker CK, Cardon G, Vandelanotte C (2015). Theory-driven, web-based, computer-tailored advice to reduce and interrupt sitting at work: development, feasibility and acceptability testing among employees. BMC Public Health.

[49] Bardus M, Blake H, Lloyd S, Suggs L (2014). Reasons for participating and not participating in a e-health workplace physical activity intervention. Intl J of Workplace Health Mgt.

[50] Koopmans L, Bernaards CM, Hildebrandt VH, van Buuren S, van der Beek AJ, de Vet HCW (2014). Improving the Individual Work Performance Questionnaire using Rasch analysis. J Appl Meas.

[51] Cohen S (1994). Perceived stress scale. Measuring stress: a guide for health and social scientists.

[52] Ware JE (2000). SF-36 health survey update. Spine (Phila Pa 1976).

[53] Klein EM, Brähler E, Dreier M, Reinecke L, Müller KW, Schmutzer G, Wölfling K, Beutel ME (2016). The German version of the Perceived Stress Scale—psychometric characteristics in a representative German community sample. BMC Psychiatry.

[54] Hagger MS, Luszczynska A, de Wit J, Benyamini Y, Burkert S, Chamberland P, Chater A, Dombrowski SU, van Dongen A, French DP, Gauchet A, Hankonen N, Karekla M, Kinney AY, Kwasnicka D, Hing Lo S, López-Roig S, Meslot C, Marques MM, Neter E, Plass AM, Potthoff S, Rennie L, Scholz U, Stadler G, Stolte E, Ten Hoor G, Verhoeven A, Wagner M, Oettingen G, Sheeran P, Gollwitzer PM (2016). Implementation intention and planning interventions in Health Psychology: recommendations from the Synergy Expert Group for research and practice. Psychol Health.

[55] McNeill LH, Kreuter MW, Subramanian SV (2006). Social environment and physical activity: a review of concepts and evidence. Soc Sci Med.

[56] Berninger NM, Ten Hoor GA, Plasqui G (2018). Validation of the VitaBit Sit-Stand Tracker: detecting sitting, standing, and activity patterns. Sensors (Basel).

[57] Fukuoka Y, Lindgren TG, Mintz YD, Hooper J, Aswani A (2018). Applying natural language processing to understand motivational profiles for maintaining physical activity after a mobile app and accelerometer-based intervention: the mped randomized controlled trial. JMIR Mhealth Uhealth.

[58] Hendriksen IJM, Bernaards CM, Steijn WMP, Hildebrandt VH (2016). Longitudinal relationship between sitting time on a working day and vitality, work performance, presenteeism, and sickness absence. J Occup Environ Med.

[59] Dunlop DD, Song J, Arnston EK, Semanik PA, Lee J, Chang RW, Hootman JM (2015). Sedentary time in US older adults associated with disability in activities of daily living independent of physical activity. J Phys Act Health.

[60] Waters LA, Galichet B, Owen N, Eakin E (2011). Who participates in physical activity intervention trials?. J Phys Act Health.

[61] Brislin RW (1970). Back-translation for cross-cultural research. J Cross-Cultural Psychol.

[62] Atkin AJ, Gorely T, Clemes SA, Yates T, Edwardson C, Brage S, Salmon J, Marshall SJ, Biddle SJH (2012). Methods of measurement in epidemiology: sedentary behaviour. Int J Epidemiol.

[63] Craig CL, Marshall AL, Sjöström M, Bauman AE, Booth ML, Ainsworth BE, Pratt M, Ekelund U, Yngve A, Sallis JF, Oja P (2003). International physical activity questionnaire: 12-country reliability and validity. Med Sci Sports Exerc.

[64] Ruiter R, Crutzen R, Kok G (2018). Core processes for developing theory-and evidence-based interventions.

[65] Smith-Dektor AH, Young SD (2014). Marketing, technology, and medicine: recommendations on how to incorporate psychological principles into new technologies to promote healthy behaviors. J Consum Health Internet.

[66] Montag C, Duke E, Markowetz A (2016). Toward psychoinformatics: computer science meets psychology. Comput Math Methods Med.

