# Supplementary figures and images for: Sedentary Work in Desk-Dominated Environments: A Data-Driven Intervention Using Intervention Mapping
Source: JMIR Form Res. 2020 Jul 20;4(7):e14951. doi: 10.2196/14951 (PMC7399954; doi:10.2196/14951)

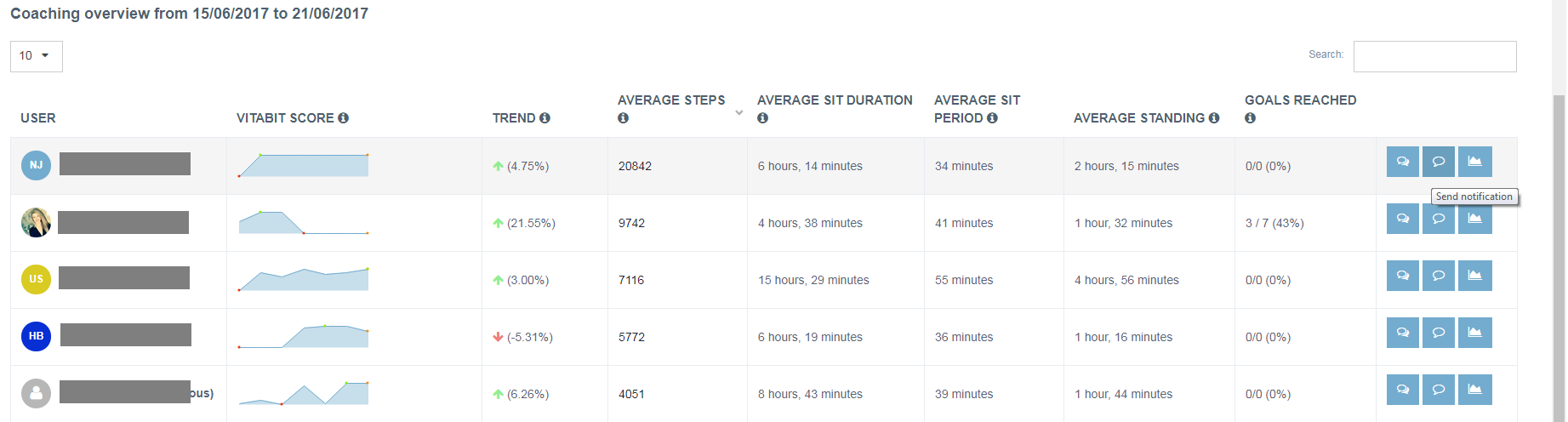

Supplement: Multimedia Appendix 1 [file formative_v4i7e14951_app1.png]
